# 1α, 25-dihydroxy Vitamin D3 containing fractions of *Catharanthus roseus* leaf aqueous extract inhibit preadipocyte differentiation and induce lipolysis in 3T3-L1 cells

**DOI:** 10.1186/s12906-019-2754-7

**Published:** 2019-11-29

**Authors:** Anuj Kumar Borah, Archana Singh, Rafika Yasmin, Robin Doley, Venkata Satish Kumar Mattaparthi, Sougata Saha

**Affiliations:** 1 Department of Molecular Biology and Biotechnology, Tezpur University, Napaam, Tezpur, Assam 784028 India; 2 Department of Biotechnology, National Institute of Technology, Durgapur, West Bengal 713209 India

**Keywords:** *Catharanthus roseus*, Obesity, Lipolysis, Adipogenesis, 3T3-L1, Vitamin D3

## Abstract

**Background:**

To investigate the potential of *Catharanthus roseus* leaf aqueous crude extract (CRACE) as a regulator of adipocyte development and function.

**Methods:**

3T3-L1 adipogenesis model was used to investigate the effect of CRACE on adipogenesis. 3T3-L1 preadipocytes (for adipogenic differentiation) and mature 3T3-L1 adipocytes (for adipocyte function) were treated with non-toxic doses of CRACE. The outcomes were corroborated by intracellular lipid accumulation, expression of pro-and anti-adipogenic effector molecules. To investigate CRACE mediated lipolysis, cAMP accumulation, glycerol release and phosphorylation of key effector molecules were tested in treated mature adipocytes. Finally, the extract was fractionated to identify the active molecule/s in the extract.

**Results:**

CRACE significantly reduced adipocyte differentiation by modulating PPARγ expression. At early stage CRACE directly targeted Lipin1 expression and consequently impacted KLF7, subsequently expression of GATA2, CEBPα, SREBP1c were targeted, with PPARγ expression, particularly curtailed. While CRACE significantly reduced several lipogenic genes like FAS and GPD1 in mature adipocytes, concomitantly, it greatly increased lipolysis resulting in decreased lipid accumulation in mature adipocytes. The increase in lipolysis was due to decreased Akt activation, increased cAMP level, and PKA activity. The fractionation of CRACE allowed identification of two fractions with potent anti-adipogenic activity. Both the fractions contained 1α, 25-dihydroxy Vitamin D3 as major component.

**Conclusions:**

1α, 25-dihydroxy Vitamin D3 containing CRACE can be developed into an effective anti-obesity formulation that decreases adipogenesis and increases lipid catabolism.

## Background

Adipose tissue development, function and its microenvironment have become potential caches to counter health issues like obesity, cardiovascular disease, type2 diabetes, cancer and many more metabolic as well as physiological disturbances [1, 2]. During adipose tissue development new fat cells arise from preadipocytes by a highly regulated process, where committed cells undergo a clonal expansion phase, followed by differentiation. PPARγ (Peroxisome Proliferator Activated Receptor γ) plays the role of a key regulator during the two-step adipogenic program, along with several other transcription factors playing positive and negative regulatory roles [3, 4]. Dysregulations of adipogenesis leads to adverse repercussions like excessive accumulation of triacylglycerol (TAG) in adipocytes (hypertrophy) and increase in the number of adipocytes (hyperplasia). Both conditions contribute to obesity; which eventually changes the tissue microenvironment, leading to recruitment of pro-inflammatory macrophages, heightened inflammation and insulin resistance [5–7]. Thus restoring homeostasis in the adipocytes is argued to be a potential therapeutic, approach that is fairly commonly adopted. Three important factors associated with adipocyte homeostasis are differentiation, lipogenesis and lipolysis. Research with adipocytes as well as obese animal models have shown that, increased lipid metabolism and decreased adipogenesis can improve glucose utilization and beneficiary adipokine expression [8]. However, at present, the compounds approved for treating obesity mostly target appetite reduction and lipid digestion, with poor efficacy and many side effects. Thus it is necessary to search for new therapeutic agents to treat obesity, which has acquired the status of a global epidemic.

Herbal extracts are proven sources of therapeutic agents with many important drugs having been derived from plant metabolites. In search of new anti-obesity therapeutic agents, the current study delved into plant extracts with potential effect on adipocytes. *Catharanthus roseus* (formerly known as *Vinca rosea*) which is locally known as nayantora/nayantara in eastern India, has many reports of its efficacy in traditional medicine against cancer, diabetes, liver disease etc. In addition, a recent study showed improved serum lipid profile after oral administration of *C. roseus* leaf juice to guinea pigs [9]. Nayantora is a bushy perennial herb of the *Apocynaceae* family, available in quite a few varieties. Its common names whether nayantara, nayantora or bright eyes, refers to characteristic darker centres or “eyes”, at the centre of its flowers that range from white to lavender pink. Hot water extract of the dried leaves has been taken for treatment of diabetes in India [10, 11], Jamaica [12], Brazil [13] and South Africa [14] among many other countries. Organic extracts of *C. roseus* are reported to have stimulatory effects on glucose utilization in 3T3-L1 cells [15]. However, no report on the effect of *C. roseus* extracts on adipose tissue development and adipocyte physiology exists. Neither the target tissues nor target molecules of this extract have been explored in depth in relation to adipocytes. In the current study, potential anti-obesity effect of *C. roseus* leaf extract was tested in an adipocyte cell culture model.

## Methods

### Collection and identification of the plant sample

Freely available *Catharanthus roseus* plant was collected from North Lakhimpur district of Assam, in India (Geographical coordinates: 27° 14′ 10.7412″ N and 94° 5′ 45.0240″ E). The plant material was further identified by Dr. S. K. Singh (Scientist-D, Botanical Survey of India, Shillong) as *Catharanthus roseus* (L.) G. Don. The voucher specimen of this material has been deposited in a publicly available herbarium repository of Botanical Survey of India, Shillong (deposition No. BSI/ERC/Tech/Plant identification/2018/276).

### Preparation of the CRACE

Leaves of the flowering plants were washed with water, shade dried and ground to powder. The powdered plant material (5 g) was mixed with 100 mL of distilled water and stirred at room temperature for 5 h. The resulting slurry was incubated for 12 h at 4 °C. The slurry was then centrifuged and the supernatant was syringe filtered to obtain the extract named “CRACE”. The liquid CRACE was further lyophilized to get the powdered CRACE. The yield of lyophilisation was 8–8.3 mg of powder per mL of liquid CRACE. The lyophilized powder was dissolved again in sterile water to obtain 10 mg/mL of CRACE stocks.

### Cell culture, differentiation and treatments

3T3-L1 cells, L6 cells, HepG2 and Raw 264.7 cells were seeded at a density of 2 X10^4^ cells/well of a 96 well plate in culture medium containing DMEM (Himedia AL007A) with 10% FBS and 1X antibiotic and antimycotic (maintenance medium) in a humidified 5% CO_2_ incubator at 37 °C. When confluency of the cells reached approximately 70%, the cells were exposed to different doses of the extract CRACE for 24 h or 48 h; subsequently, and cell survivability was measured by MTT (Sigma M2128) assay following standard procedure [16, 17]. 3T3-L1 cells were differentiated following previously reported method with minor modifications [18, 19]. Briefly, 3T3-L1 cells were seeded (3 X 10^5^ cells/well of a 35 mm dish) in maintenance medium and allowed to grow till 100% confluency. Differentiation was induced in confluent cells by replacing maintenance media with fresh maintenance media containing 1 μM dexamethasone (Sigma D4902), 0.5 mM Isobutylmethylxanthine (IBMX) (Sigma I7018) and 5 μg/mL (872 nM) Insulin (Sigma I5500) (differentiation media). The day of induction is marked as ‘day 0’. On day 2, the media was replaced with fresh maintenance media containing only 5 μg/mL (872.07 nM) of insulin and cells were incubated for 2 days. On day 4 the media was replaced with maintenance media and cells were grown for another 4 days to allow them to differentiate. By day 8, the 3T3-L1 preadipocytes had become adipocytes with accumulated lipid droplets. To test the effect of CRACE and 1α, 25-dihydroxy Vitamin D3 (Sigma D1530) on differentiation, cells were exposed to the extract or the compound from day 0 to day 4. To test the effect of CRACE and 1α, 25-dihydroxy Vitamin D3 on mature adipocytes, the extract or the compound was added to the differentiated adipocytes on day 8, and cells were grown for another 4 days, with change of medium every 2 days, unless mentioned otherwise. For expression analysis (mRNA and protein), and cell based assays (viz cAMP accumulation, glycerol release assay and lipid droplet size study), the liquid CRACE was used and represented as the μg/mL equivalence of the powdered CRACE. The treatments for lipid accumulation study, during differentiation and post differentiation, were done with different concentrations of lyophilized powered CRACE, from the 10 mg/mL CRACE stocks.

### Oil red O staining and quantification of lipid content and lipid droplet size

Accumulated intracellular lipid was stained with Oil Red O (ORO) as described before with few modifications [19]. Briefly, cells were stained with 3:2 of 0.3% ORO in isopropanol and water for 5–10 min followed by three 60% isopropanol washes, and three water washes. Lipid content was measured by extracting ORO by isopropanol and measuring absorbance at 510 nm. Lipid content was also measured by analyzing ORO fluorescence per frame of micrographs of stained cells by ImageJ software from NIH following earlier report [20]. To measure lipid droplet size, phase contrast and fluorescent micrographs of ORO stained cells were captured at 100X. Area of all lipid droplets from 5 individual images of ORO stained cells were measured using area measurement module of NLS element analysis D software.

### RNA isolation and semi quantitative RT PCR

Total RNA was isolated from cultured cells using TRIzol (Invitrogen) and cDNA synthesis was done using cDNA synthesis kit (Clontech, Otsu, Japan; 6110A). Specific primer sequences (Additional file 1: Table S2) were used to amplify target genes. Intensities of bands were quantified by GelQuant.NET software (Biochemlabsolutions, Wayne, PA, USA).

### Western blotting

Anti-PPARγ was purchased from Thermo Scientific (MA5–14889); whereas anti-β-Tubulin Ab (AB0119) and anti-γ-Actin antibody (BB-AB0025) were purchased from BioBharati, India. Anti-PLN1, Anti-PKA substrate specific antibody (Anti pPLN1 antibody), Anti-AKT and Anti- pAKT(S473) were obtained from CST (CST 9349, 9621, 9272S and 4060 T respectively). Blots were developed using West Femto (Invitrogen 34,094) or Clarity ECL substrate (Bio-Rad 1,705,060) and documented using chemidoc.

### Glycerol release assay

3T3-L1 (7.5X10^4^ cells/well) were seeded in 96 well plates and allowed to differentiate into mature adipocytes. On day 8 post differentiation, cells were transferred to Krebs Ringer Buffer (KRB buffer) with 4% fatty acid free BSA and 5 mM D-glucose (lipolysis buffer). Lipolysis was induced by either 10 μM isoproterenol (Sigma I6504) or CRACE (500 μg/mL equivalent) in lipolysis buffer and incubated for 3 h in CO_2_ incubator. Similar treatments were also done after treating cells for 1 h with 10 μM adenosine (Sigma A4036-5G) in lipolysis buffer. Subsequently, 3 h of isoproterenol or CRACE treatment in presence of 1 U/mL adenosine deaminase (Sigma 52,544-1ML) was initiated to remove adenosine mediated background inhibition [21]. To test the effect of PKA inhibition on CRACE induced lipolysis, mature adipocytes were serum starved for 3 h. The cells were then pre-treated with 20 μM PKA inhibitor H89 (Tocris 2910) for 1 h in lipolysis buffer, followed by 3 h treatment with 500 μg/mL equivalent CRACE or 10 μM isoproterenol individually or together in presence of 20 μM H89. Uninduced and no treatment groups were taken as control. At the end of the treatments released glycerol was estimated using glycerol assay kit (Sigma MAK117).

### cAMP accumulation assay

3T3-L1 (2X10^4^ cells/well) were plated in white clear bottom 96 well plates (BD 353377) and grown overnight. Cells were serum starved for 3 h. Cells were transferred to 100 μL KRB buffer with 4% fatty acid free BSA and pre-treated with 0.5 mM IBMX for 10 min followed by 15 min and 100 min of IBMX± CRACE (500 μg/mL equivalent) treatment. Intracellular cAMP was measured using cAMP-Glo Kit (Promega V1501).

### Phytochemical analysis

Identification of phytochemicals in the extract was performed following earlier reported methods. Briefly test for phenolics (ferric chloride test, phosphomolybdic acid test), flavonoids (NaOH test, Zn-HCl test, Shinoda test), saponins (foam test), tannins (Braemer’s test), steroids (Salkowski test), reducing (Fehling test) and non-reducing sugars (Iodine test) were performed [22].

### HPLC fractionation of CRACE

Reverse-Phase UHPLC was performed in Thermo Scientific Ultimate 3000, to fractionate the extract CRACE, in a Phenomenex (Aeris Widepore) C18 column (3.6 μ, 200 Å, 250*2.10). The loading volume was 200 μL of 10 mg/mL CRACE. Flow rate maintained at 0.3 mL/min. Solvent A was Milli Q water + 0.1% Trifluoroacetic acid (TFA) and Buffer B was 80% Acetonitrile (ACN) + 0.1% TFA+ Milli Q water. The separation was done with the set up as: 0–9 min, 0% Buffer B; 9–12 min, 5% Buffer B; 12–42 min, 30% Buffer B; 42–48 min, 100% Buffer B; 48–54 min, 100% Buffer B; and finally 54–66 min, 0% Buffer B. Separated peaks were monitored at 254 nm and 280 nm wavelengths and collected in sterile tubes. The material was dried by evaporation and collected peaks were dissolved with 200 μL of sterile Milli Q water and stored at − 20 °C until further use. Compositions of the small molecules from the fractions were identified by HR-LCMS analysis and library search at IIT, Bombay SAIF Facility. HPLC was performed using a Hypersil Gold (3 μ) 100 * 2.1 mm column. The abundance of the molecules was estimated from the heights of the peaks. The drug-likeness behaviour (in accordance with Lipinski rule) of molecules present in CRACE was calculated using MolSoft Drug-likeness tool.

### Statistical analysis

Statistical significance (*p* value) was calculated by two tailed unpaired t-test or one-way ANOVA or two-way ANOVA followed by Bonferroni post-test using Graph Pad Prism as mentioned in the figure legends.

## Results

### CRACE reduced lipid accumulation in differentiating 3T3-L1 cells

**Fig. 1:**
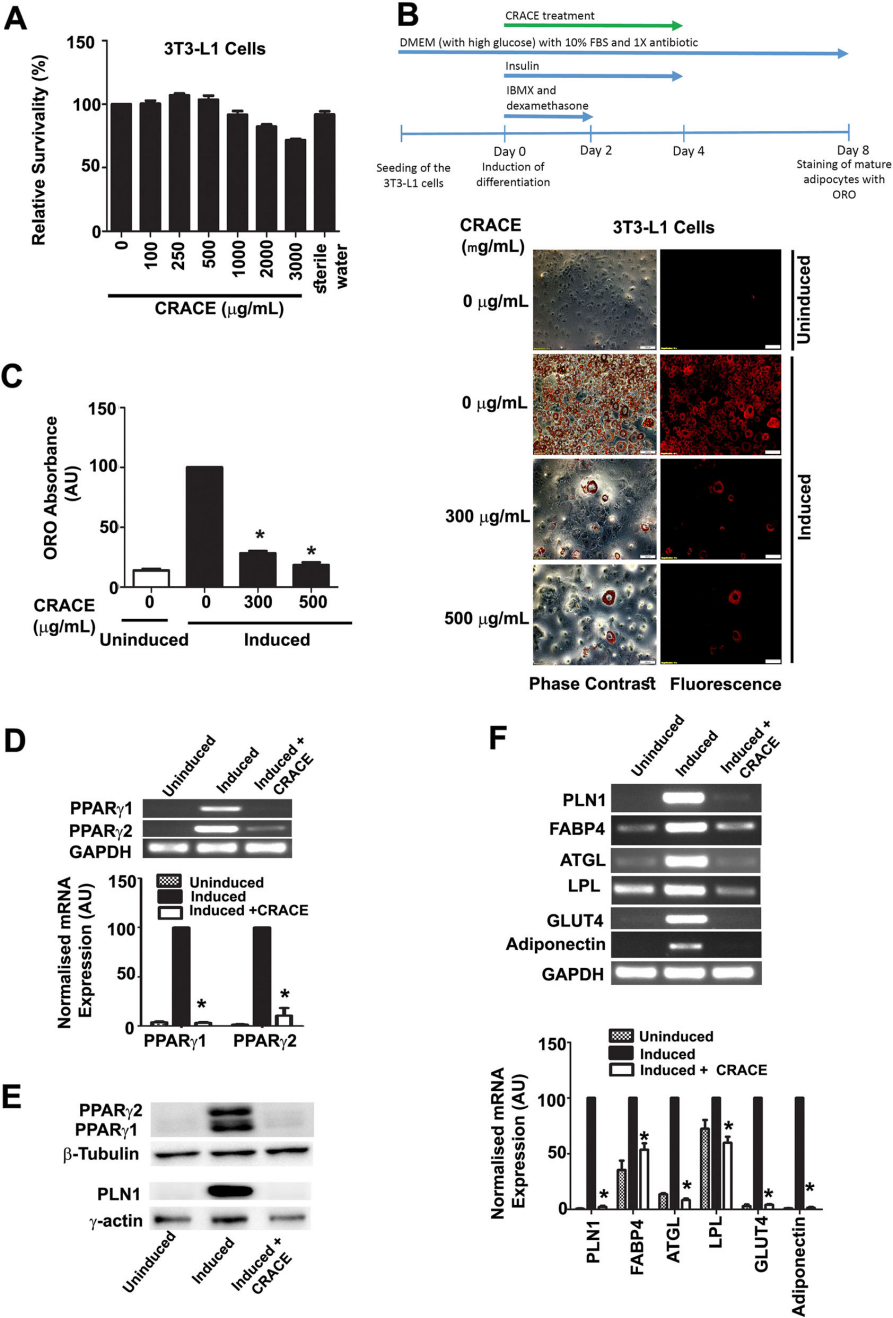
CRACE inhibited differentiation of 3T3-L1 preadipocytes. **a.** Percent survivability of 3T3-L1 cells as measured by MTT assay after exposure to different doses of CRACE for 24 h. **b.** CRACE reduced lipid accumulation in differentiating 3T3-L1 cells. Top panel represents the design of the experiment. 3T3-L1 cells were induced with adipogenic cocktail ± CRACE at the displayed doses for 4 days and subsequently kept in maintenance medium for an additional 4 days. Thereafter, ORO staining was performed. Images represent micrographs of ORO stained cells. Scale bar: 100 μm. **c.** Graph represents absorbance of extracted ORO from different groups of cells represented in panel **b**. **d.** Expression of PPARγ1 and PPARγ2 transcripts were reduced in CRACE treated group of induced 3T3-L1 cells. 3T3-L1 cells were induced for differentiation ± CRACE (500 μg/mL equivalent) in the same way as described in panel **b**. Gel images and bar diagram represent RT-PCR analysis of total RNA isolated after 8 days of induction. **e.** CRACE reduced PPARγ1, PPARγ2 and PLN1 protein level in differentiating 3T3-L1 cells are represented in panel **a**. Gel pictures represent western blot analysis of proteins isolated after 8 days of induction. **f.** CRACE reduced expression of PPARγ target genes**.** Cell differentiation, and treatment is the same as in panel **d**. Gel images and bar diagram represent RT-PCR analysis of PPARγ regulated adipocyte factors: PLN1, FABP4, ATGL, LPL, GLUT4, and Adiponectin at 8 days after induction. All bars in the figure represent mean ± SEM, *n* = 3. *p* values are from unpaired t-test. * indicates *p* < 0.05

Prior to test the effect of CRACE on adipocyte differentiation, nontoxic dose of the extract was determined by exposing 3T3-L1 cells to different doses of the extract for 24 h followed by MTT Assay (Fig. 1a). Up to 1000 μg/mL CRACE treatment was found to be nontoxic. The extract was found to be nontoxic to other tested cell types namely: mature adipocytes, muscle cells, hepatocytes and macrophages (Additional file 1: Figure S1). To determine the composition of CRACE, a preliminary phytochemical assay was performed, and presence of phenolics and tannins were detected in the extract. Total phenolic, flavonoid and tannin content were found to be 54.6 μg Gallic acid equivalent, 26.5 μg Quercetin equivalent, and 392.6 μg equivalent of Tannic acid/mg, of the lyophilized CRACE respectively (Additional file 1: Table S1).

To test the effect of the extract on preadipocyte differentiation, 3T3-L1 cells were induced to differentiate in the presence of 300 μg/mL and 500 μg/mL CRACE. Oil Red O staining of the intracellular lipids at 8 days of post induction revealed that, CRACE inhibited lipid accumulation in differentiating cells at both the doses, as confirmed by elution of Oil Red O from stained cells (Fig. 1b and c).

### CRACE modulated adipogenesis by modulating expression of key regulatory factors

Transcription factor PPARγ has been at the center of the adipogenic program; required for expression and activation of factors necessary for lipid accumulation, insulin sensitivity as well as lipid metabolism [23, 24]. Among two PPARγ isoforms, while PPARγ1 is expressed in varying amounts in a wide array of cells, PPARγ2 expression is limited to only adipose tissue [25, 26]. Estimation of PPARγ mRNAs and protein at the end of 8 days of differentiation showed that CRACE targeted both the isoforms of PPARγ. Expression of both the PPARγ isoforms were suppressed during differentiation (Fig. 1d and e).

Differentiated adipocyte markers PLN1, FABP4, LPL, ATGL, GLUT4, and adiponectin, are downstream targets of PPARγ, which govern the processes of lipid accumulation, glucose uptake, and lipid metabolism in differentiating adipocytes [23]. Analysis of these genes after 8 days of differentiation showed treatment with CRACE, downregulated all these PPARγ target genes, leading to inhibition of adipocyte differentiation (Fig. 1f). PLN1 protein content was also drastically reduced in CRACE treated differentiating 3T3-L1 cells (Fig. 1e).

### CRACE modulated expression of early and late adipogenic regulators

**Fig. 2:**
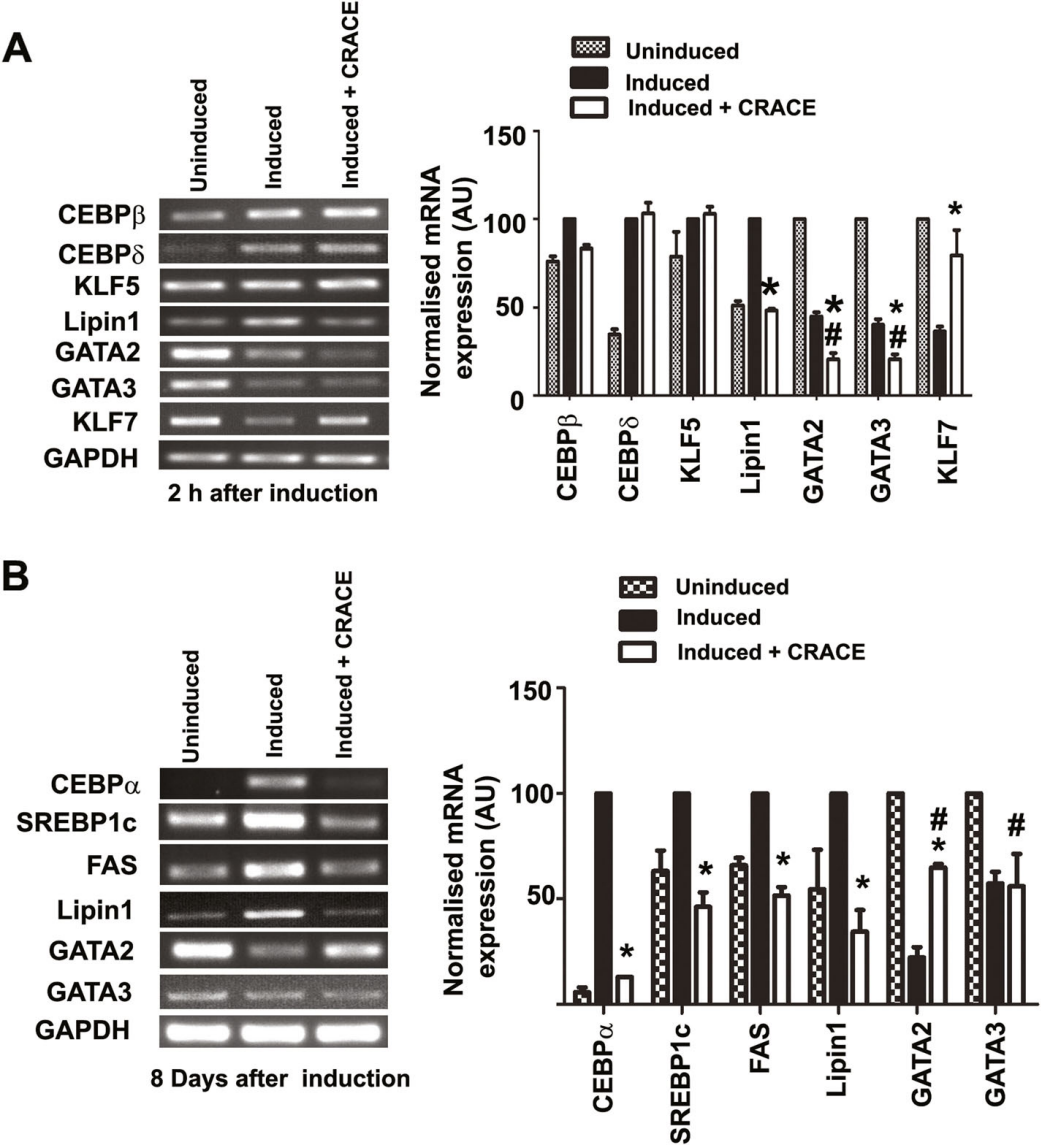
CRACE modulated expression of pro- and anti-adipogenic regulators at early and late stage of adipogenesis. **a**. Gel images and bar diagram/diagrams represent RT-PCR analysis of early adipogenic regulators expressions in presence and absence of CRACE (500 μg/mL equivalent); after 2 h of induction of adipogenesis in 3T3-L1 cells. **b**. Gel images and bar diagram represent RT-PCR analysis of adipogenic regulators expressions in presence and absence of CRACE (500 μg/mL equivalent); at the late stage of adipogenesis (8 days post adipogenesis induction in 3T3-L1 cells). Bars represent mean ± SEM. *n* = 3. GAPDH mRNA expression was used as internal control. Unpaired t-test was performed to study the statistical significance. * represents *p* < 0.05 for “Induced” vs “Induced + CRACE”. # represents *p* < 0.05 for “Uninduced” vs. “Induced + CRACE”

**Fig. 3:**
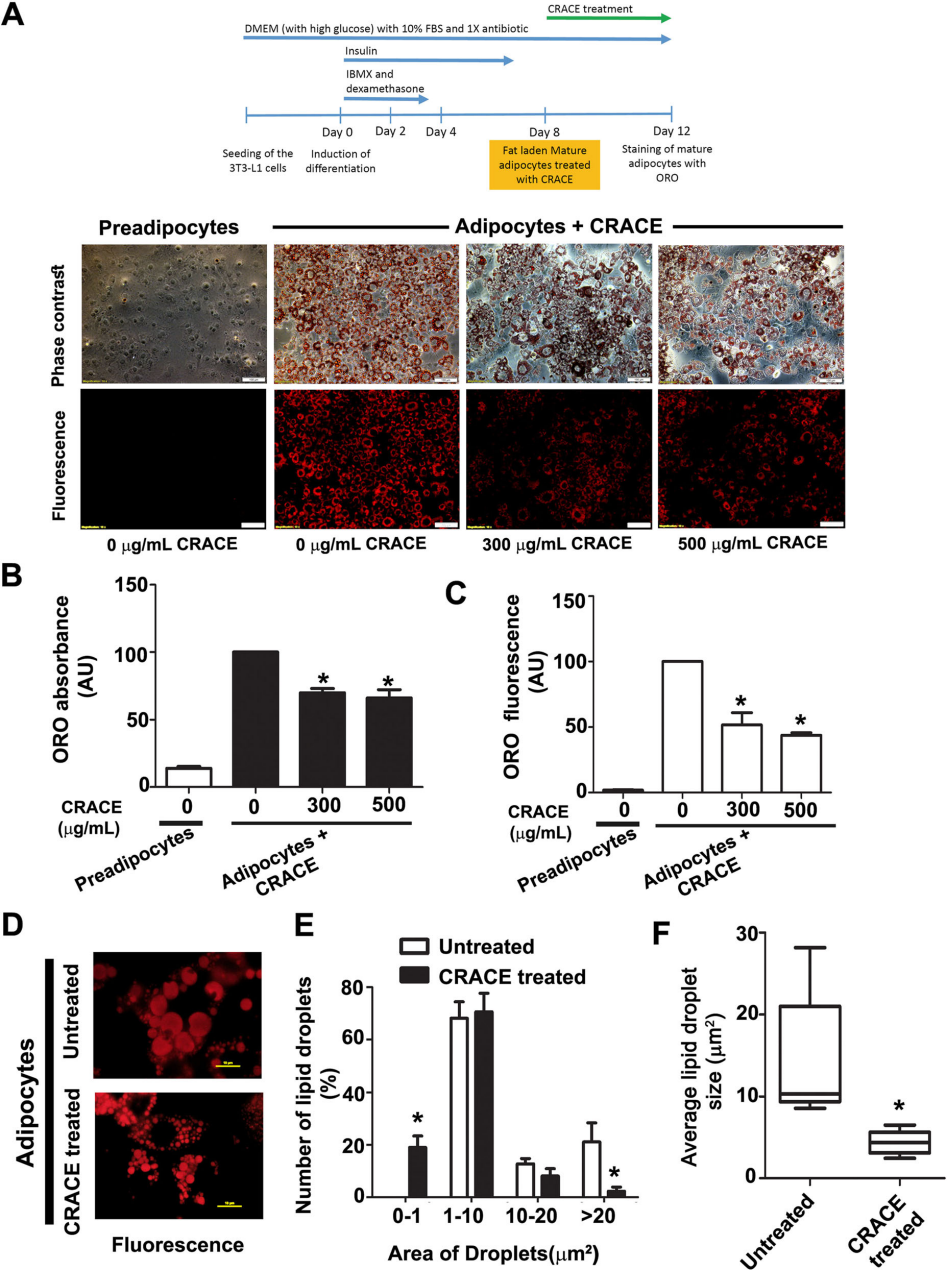
CRACE decreased lipid accumulation and lipid droplet size in mature adipocytes. 3T3-L1 cells were differentiated to mature adipocytes followed by treatment with CRACE (500 μg/mL equivalent) for 4 days. Lipid content was estimated by ORO staining. **a**. Top panel represents design of the experiment. Micrographs represent ORO stained preadipocytes and CRACE treated and untreated mature adipocytes at doses of 300 and 500 μg/mL. Scale bars: 100 μm. **b** and **c**. Quantification of lipid in cells shown in panel **a** by ORO elution (**b**) and by measuring total ORO fluorescence per micrograph frames (**c**). Bars represent mean ± SEM. For panel **b**, *n* = 3. For panel **c**, *n* = 10 image frames. Unpaired t-test was performed to study the statistical significance. * *p* < 0.05 for untreated vs. CRACE treated. **d**-**f**. CRACE decreased size of lipid droplets in mature adipocytes. **d**. High magnification fluorescent micrographs of ORO stained untreated and CRACE treated mature adipocytes are shown in panel **a**. Scale bars: 10 μm. **e**. Population distribution of small to large size lipid droplets in the cells shown in panel **d**. Bars represent mean ± SEM. *n* = 5 image frames. * *p* < 0.05 for untreated Vs CRACE treated. One-way ANOVA followed by Bonferroni post-test was performed to evaluate the statistical significance. **f**. Average lipid droplet size calculated from the data represented in panel **e**. Box and whisker (minimum to maximum) representation of lipid droplet sizes in each group. Unpaired t-test was performed to study the statistical significance. * *p* < 0.05 for untreated Vs CRACE treated

To understand the mechanism of CRACE mediated PPARγ downregulation, mRNA expression of key transcription regulators of PPARγ gene were analyzed at the early stage of differentiation (2 h post induction). CEBPβ and CEBPδ expression occur very early during adipogenesis, and are the main inducers of PPARγ gene [27]. KLF5 is a pro-adipogenic molecule, which is expressed within an hour of adipogenic induction, enhancing the expression of PPARγ2 [28, 29]. At a downstream point, CEBPα is a key cross regulator of PPARγ expression, and these two genes regulate each other’s expression [30]. Lipin1 is expressed at early point of differentiation and can increase PPARγ expression [31]. There are several negative regulators of adipogenesis like GATA2, GATA3, and KLF7 which need to be downregulated during preadipocyte differentiation. While at early point of adipogenesis GATA2 and GATA3 interact with CEBPβ and represses its expression [32], at a later stage GATA2 and GATA3, are reported to interact with CEBPα repressing PPARγ expression [33]. The other anti-adipogenic factor, KLF7, expression reduces PPARγ, CEBPα, FABP4, Adipsin etc. [34]. Expression analysis of these genes at the early stage of differentiation revealed that CRACE did not affect the expression of CEBPβ, CEBPδ, KLF5, GATA2 and GATA3 (Fig. 3a). However, it did affect the expression of a positive regulator Lipin1 and was a distinct negative regulator of KLF7 (Fig. 2a).

Gene expression analysis of adipogenic regulators at the later stage of differentiation (8 days post induction), revealed down regulation of key positive regulator of PPARγ expression, CEBPα, Lipin1 and other similar positive regulators like SREBP1c and FAS in the extract treated cells (Fig. 2b). SREBP1c, an integral component of lipid biosynthesis, is reported to activate PPARγ as well as provide ligands for PPARγ [35]; and is an up-regulator of GLUT4 expression in adipocytes [36]. FAS is another PPARγ regulator reported to be under influence of SREBP1 activity [37–39]. The anti-adipogenic effect of the extract was further strengthened by the loss of activity of the repressor GATA2 in the treated cells with no concomitant variation in the negative regulator GATA3 expression (Fig. 2b).

### CRACE reduced fat accumulation in mature adipocytes

To test if CRACE can reduce lipid content of mature adipocytes, completely differentiated 3T3-L1 adipocytes with equal initial lipid droplets, were treated with CRACE at different doses for 4 days. ORO staining of the cells showed CRACE could reduce lipid accumulation in mature adipocytes, at all the tested doses (300 μg/mL and 500 μg/mL) in a dose dependent manner (Fig. 3a). Quantification of lipid in treated and untreated cells by ORO extraction (Fig. 3b), and ORO fluorescence in microscopic images (Fig. 3c), showed > 50% drop in lipid accumulation in treated cells. Further comparison of lipid droplets in untreated vs CRACE (500 μg/mL equivalent) treated mature adipocytes, showed that the average lipid droplet area was 14.2 ± 3.6 μm^2^ in untreated cells, and 4.4 ± 0.7 μm^2^ in treated cells (Fig. 3d and f). Moreover, number of lipid droplets > 20 μm^2^ was significantly low in CRACE treated adipocytes, while the number of lipid droplets < 1 μm^2^ was significantly low in CRACE untreated group of adipocytes, suggesting reduction in lipid accumulation in lipid droplets (Fig. 3e).

### CRACE reduced lipogenesis in 3T3-L1 adipocytes

**Fig. 4:**
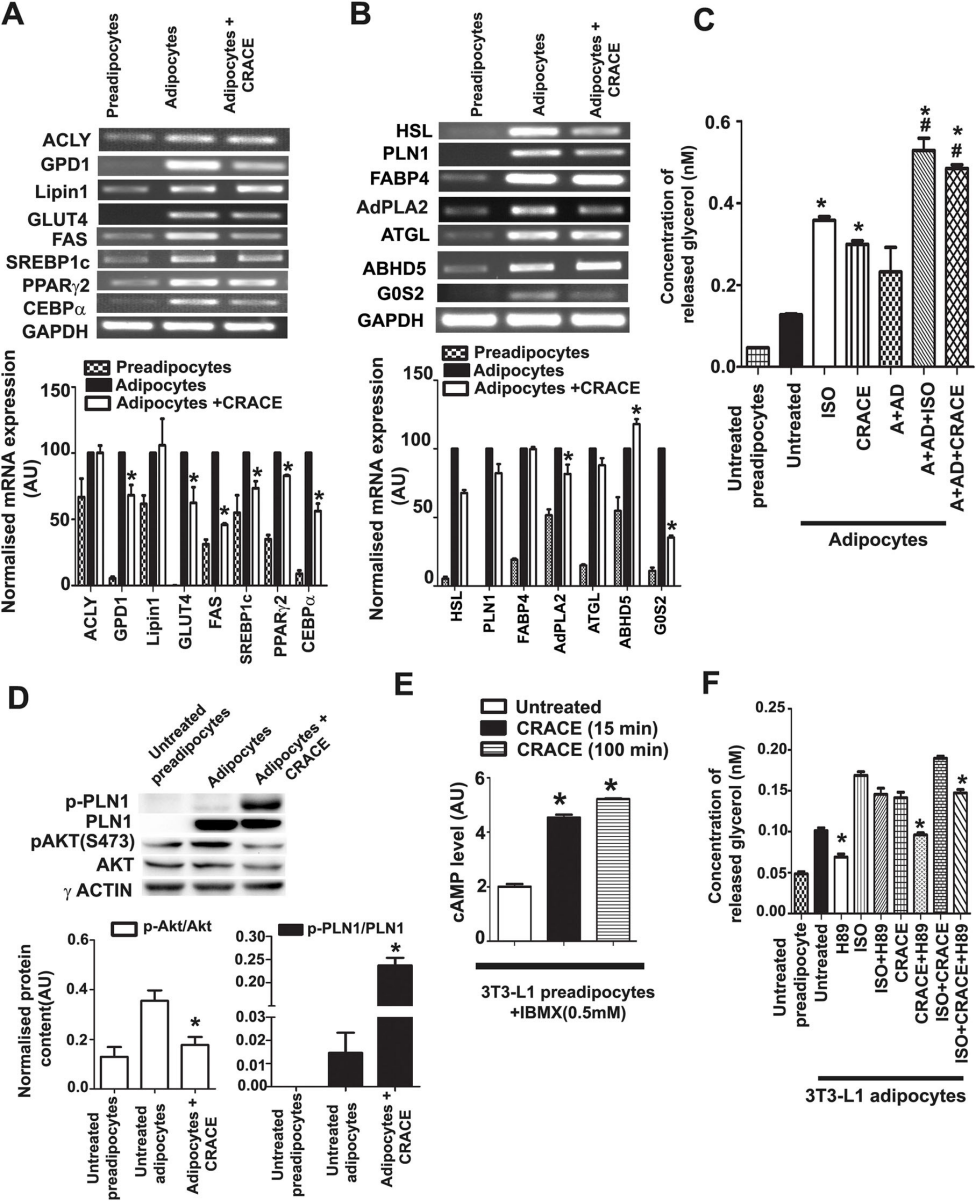
CRACE reduced lipogenesis and induced lipolysis in mature 3T3-L1 adipocytes by increasing cAMP level and PKA activation. **a**. mRNA expression analysis of lipogenesis related genes in CRACE treated and untreated mature adipocytes. **b**. mRNA expression analysis of major players of TAG lipolysis in CRACE treated and untreated mature adipocytes. In panel **a** and **b** mature adipocytes received CRACE (500 μg/mL equivalent) for 4 days. Bars represent mean ± SEM. *n* = 3. * *p* < 0.05 for untreated vs. CRACE treated. **c**. Treatment with CRACE (500 μg/mL equivalent) for 3 h increased lipolysis in mature 3T3-L1 adipocytes as measured by glycerol release. Isoproterenol (10 μM) was used as positive control. CRACE also increased lipolysis in cells where basal lipolysis was normalised with 100 nM adenosine (A) and 1 U/mL of adenosine deaminase (AD) treatment. Bars represent mean ± SEM. *n* = 3. * indicates *p* < 0.05 for untreated vs. different treatments and # indicates *p* < 0.05 for A + AD vs. A + AD + different treatments. **d**. CRACE increased PLN1 phosphorylation and decreased Akt phosphorylation (S473). Mature 3T3-L1 adipocytes were treated with CRACE (500 μg/mL equivalent) for 24 h followed by western blot analysis. Bars represent mean ± SEM, *n* = 3. * *p* < 0.05 for untreated vs. CRACE treated adipocytes. **e**. CRACE (500 μg/mL equivalent) increased intracellular cAMP level in mature 3T3-L1 adipocytes. 0.5 mM IBMX was added in all the cells for cAMP sustenance. Bars represent mean ± SEM, *n* = 3. * *p* < 0.05 for untreated vs. treated adipocytes. **f**. CRACE stimulated lipolysis by PKA activation in mature 3T3-L1 adipocytes. Glycerol release induced in mature adipocytes by CRACE (500 μg/mL equivalent) or isoproterenol in presence or absence of PKA inhibitor H89 was measured. CRACE induced lipolysis was significantly reduced by H89. Bars represent mean ± SEM, *n* = 3. * *p* < 0.05 for ‘given treatments’ vs. ‘treatment + H89’ (i.e. Untreated vs H89; CRACE vs CRACE + H89; ISO + CRACE vs ISO + CRACE + H89). Unpaired t-test (for panel 4**a**, 4**b**, 4**d** and 4**e**) and one-way ANOVA followed by Bonferroni post-test (for panel 4**c** and 4**f**) was performed to evaluate the statistical significance

To test if reduced lipogenesis was the reason of reduced lipid accumulation in CRACE treated adipocytes, mRNA expression of factors related to these pathways were tested. PPARγ, CEBPα, FAS and SREBP1c are factors that regulate lipogenesis in differentiated 3T3-L1 cells [40, 41]. While PPARγ and CEBPα expression showed moderate decrease in CRACE treated cells, SREBP1c and FAS expression were decreased to a significant level (Fig. 4). While expression of some of the enzymes involved in lipid synthesis, and lipid droplet biogenesis, like ATP citrate lyase (ACLY) and Lipin1 were not affected by CRACE, the extract reduced expression of other important enzymes like Glycerol Phosphate Dehydrogenase 1 (GPD1) and FAS significantly (Fig. 4a). Expression of major glucose transporter GLUT4 was somewhat reduced by the extract. These observations suggested that lipogenesis may be affected to a certain extent in CRACE treated mature adipocytes.

### CRACE induced lipolysis by inducing cAMP level and PKA activation

To test the extract’s effect on lipolysis pathway, mRNA expression of several of the regulatory genes and enzymes were analysed (Fig. 4b). While major triacylglycerol (TAG) lipase ATGL expression was not changed by CRACE, its activator ABHD5 showed minor increased expression in CRACE treated cells. Expression of the major diacylglycerol (DAG) lipase, HSL, was moderately decreased in CRACE treated cells. However, at the protein level, HSL activity depends on its own phosphorylation and the phosphorylation of PLN, which coats the lipid droplet DAG [42]. Phosphorylated HSL is brought to phospho-PLN-DAG by the FABP4 molecules [43]. The activity of ATGL also depends on PLN phosphorylation [44]. Expression of both, PLN and FABP4, were found to be unchanged in CRACE treated cells (Fig. 4b). Interestingly expression of G0S2 (G0/G1 Switch Gene 2), a negative regulator of ATGL activity, was decreased to a great extent upon CRACE treatment, implied a possible increase in ATGL activity (Fig. 4b) [45].

To test if there is any increase in lipase mediated lipolysis in CRACE treated cells, glycerol release was measured in treated and untreated cells (Fig. 4c). Treatment of fully differentiated fat cells with CRACE (500 μg/mL equivalent) and known lipolysis inducer isoproterenol (10 μM) for 3 h showed 2.4-fold increase in glycerol release in CRACE treated cells. This was comparable to isoproterenol treated cells which showed 2.8-fold increase in released glycerol. When the same assay was conducted with background lipolysis, balanced by treating cells with adenosine, followed by adenosine deaminase to remove adenosine mediated inhibition of lipolysis [46], similar induction of lipolysis in CRACE and isoproterenol treated cells were observed. Glycerol release was estimated to be 2.1 fold in CRACE and 2.3 fold in isoproterenol treated cells compared to untreated adipocytes (A + AD).

PKA dependent phosphorylation of HSL and PLN is essential for induction of lipolysis. While CRACE did not affect overall PLN1 level, it significantly increased p-PLN1 level in adipocytes (Fig. 4d) indicating PKA activation. Interestingly CRACE decreased activating phosphorylation of Akt (S473) in adipocytes (Fig. 4d), which can lead to PKA activation by increasing cAMP accumulation. As PKA activation requires cAMP, its level was measured in CRACE treated 3T3-L1 preadipocytes. CRACE (500 μg/mL equivalent) treatment certainly increased cAMP level in 3T3-L1 preadipocytes > 2-fold within 15 min of treatment (Fig. 4e). A moderate decrease in expression of AdPLA2, which inhibited cAMP production and accumulation [33], was also observed in mature adipocytes, after treatment with CRACE (500 μg/mL equivalent) for 4 days (Fig. 4b). Finally effect of PKA inhibitor H89 on CRACE induced lipolysis was tested [47]. H89 could inhibit CRACE induced lipolysis significantly (Fig. 4f). However, isoproterenol induced lipolysis was not reduced to an equivalent level, as it is not dependent on PKA mediated activation of HSL and PLN1 [48]. The synergistic effect of CRACE and isoproterenol in lipolysis, was also lowered by H89 suggesting activation of PKA in presence of CRACE (Fig. 4f). All these observations suggested that CRACE induced lipolysis is due to increased cAMP level and activation of PKA.

### Presence of potent anti-adipogenic factor 1α, 25-dihydroxy vitamin D3 in most active fractions of CRACE

**Fig. 5:**
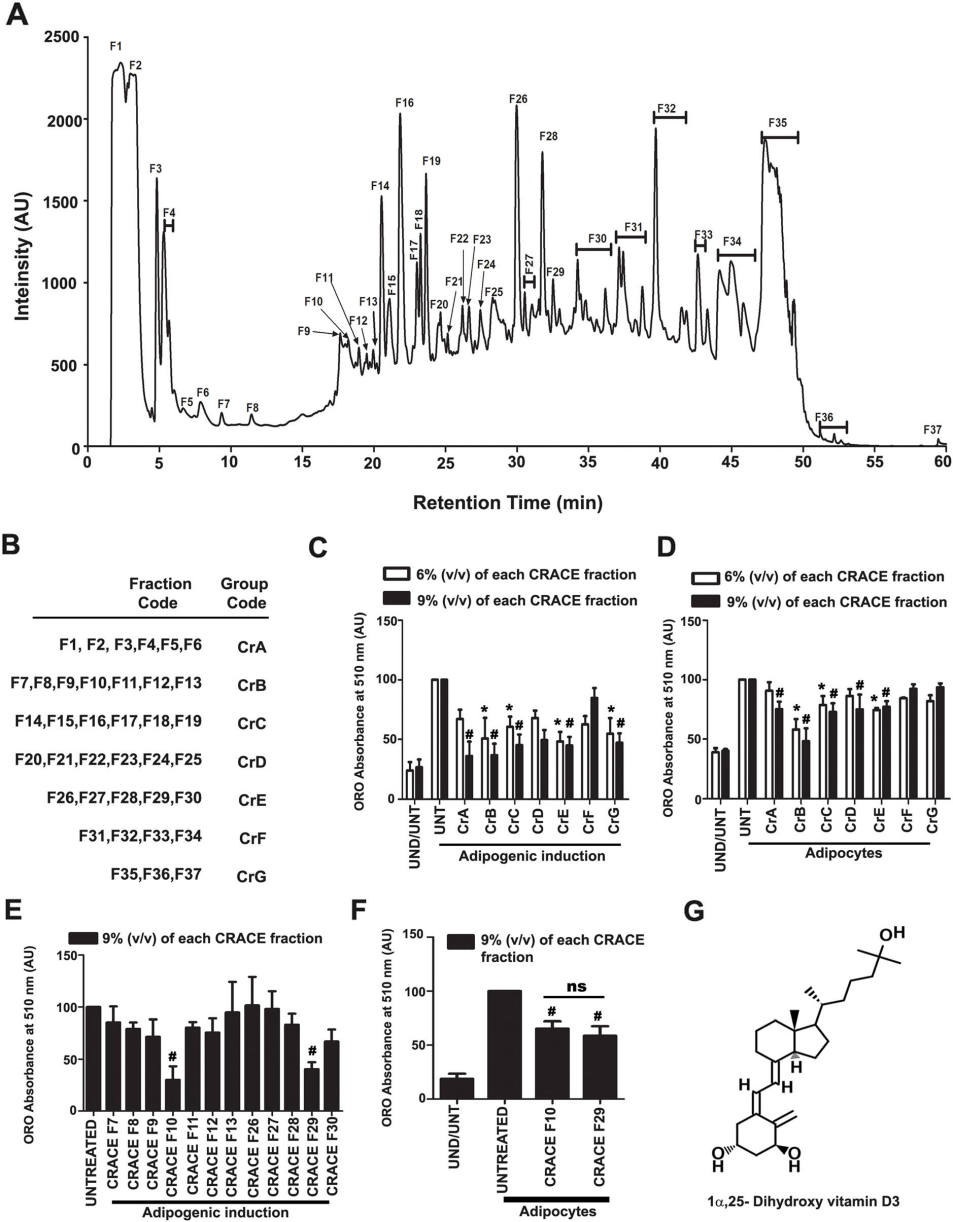
1α, 25-dihydroxy vitamin D3 was present in most active fractions in CRACE. **a**. HPLC Chromatogram of fractionation of CRACE. 200 μL of 10 mg/mL of CRACE was fractionated and later the dried fractions were dissolved again with 200 μL of sterile milli Q water. **b**. Fractions were grouped into 7 preparations named as CrA, CrB, CrC, CrD, CrE, CrF and CrG. **c**. CRACE fraction groups CrB and CrE decreased 3 T3-L1 differentiation most efficiently. The cells received treatments with all the grouped CRACE fractions (at 6 and 9% v/v for each fraction) for first 4 days of adipogenic induction. Thereafter, the cells were allowed to be maintained for another 4 days before ORO staining was performed. Bars represent mean ± SEM, *n* = 3. Two-way ANOVA followed by Bonferroni post-test was performed to evaluate the statistical significance. * indicates *p* < 0.05 for induced-untreated (UNT) vs. induced- 6% treated. # represents *p* < 0.05 for induced-untreated (UNT) vs. induced- 9% treated groups. **d**. Fraction group CrB inhibited lipid accumulation in mature adipocytes most efficiently. Mature adipocytes with equal amount of lipid received treatment with all the grouped fractions (6 and 9% v/v) for 4 days. Post treatment lipid content was measured by ORO staining. Bars represent mean ± SEM, *n* = 3. Two-way ANOVA followed by Bonferroni post-test was performed to evaluate the statistical significance. * and # indicate same as panel **c**. **e**. All the fractions in group **b** and **e** were tested in the same manner as C at 9% v/v for their effect on adipocyte differentiation. Fraction F10 and F29 appeared as active fractions in CrB and CrE respectively. Bars represent mean ± SEM, *n* = 3. One-way ANOVA followed by Bonferroni post-test was performed to the evaluate statistical significance. # indicates same as panel **c**. **f**. Fraction F10 and F29 were tested in the same manner as D at 9% v/v for their effect on reduction of fat accumulation in mature adipocytes. Both the fractions reduced accumulated lipid in mature adipocytes. Bars represent mean ± SEM, *n* = 3. One-way ANOVA followed by Bonferroni post-test was performed to evaluate the statistical significance. # indicates same as panel **c**. **g**. 1α, 25-dihydroxy vitamin D3 is the common molecule which is present in F10 and F29 as major constituent

To identify the component in CRACE which was responsible for its anti-adipogenic effect, 2 mg lyophilized extract was fractionated by reverse-phase HPLC. Single or combinations of peaks were collected in 37 fractions. Each fraction was dried before dissolving with 200 μL of water (Fig. 5a). Fractions were combined in 7 groups (CrA to CrG) (Fig. 5b) and were tested for their effect on adipocyte differentiation in the same manner as described in Fig. 1b and c. Cells were treated with grouped fractions in such a way that it equaled 6 and 9% v/v for each fraction. Considering both the doses, two groups CrB and CrE, showed best inhibitory effect (Fig. 5c). Similarly, the grouped fractions were tested for their effect on adipocyte maturation process in the same manner as given in Fig. 3a and b. In this assay only group CrB showed potent inhibitory effect (Fig. 5d). To identify the fraction/s in these groups which contained the anti-adipogenic activities, individual fractions in these two groups were tested for their inhibitory effect on adipocyte differentiation. Fraction F10 and F29 appeared as active fractions in CrB and CrE respectively (Fig. 5e). These two fractions also showed equal inhibitory effect on adipocyte maturation process (Fig. 5f). To determine the composition of the small molecules in fractions F10 and F29, HR-LCMS analysis of the fractions were performed (Table 1). One common and abundant molecule with potent anti-adipogenic effect in both the fractions turn out to be 1α, 25-dihydroxy vitamin D3, which is the biologically active form of vitamin D3 (Fig. 5g). Interestingly F10, the fraction which contains a higher percentage of the molecule, inhibited adipocyte differentiation more effectively. Apart from 1α, 25-dihydroxy vitamin D3, both the fractions also contained significant amounts of a few other compounds. Many of these molecules including 1α, 25-dihydroxy vitamin D3 are potential drug like molecules as suggested by their drug likeliness score (Table 1).

**Table 1:** List of abundant small molecules present in CRACE fraction F10 and F29 and their relative abundance observed by HR-LCMS analysis

Small Molecules	Molecular formula	Rel. Abundance (%)	Drug Likeness Score
CRACE F10			
1α, 25 - dihydroxy Vitamin D3	C_27_H_44_O_3_	34.4	0.47
Indole acrylic acid	C_11_H_9_NO_2_	12.3	−1.75
Promazine sulfoxide	C_17_H_20_N_2_OS	9.3	0.98
Dehydroprotenone	C_23_H_20_O_6_	7.0	−0.35
Grayanotoxin1	C_22_H_36_O_7_	6.4	−0.39
Ranitidine	C_13_H_22_N_4_O_3_S	6.0	1.00
N-(4-benzenesulfonamide) arachidonoyl amine	C_26_H_38_N_2_O_3_S	4.9	0.08
9,12-octadecadienal	C_18_H_32_O	3.1	−1.27
7-aminonitrazepam	C_15_H_13_N_3_O	3.0	0.30
Griseofulvic acid	C_16_H_15_ClO_6_	2.9	−0.06
N-(1R-methyl-2-hydroxy-ethyl)α, α, dimethyl arachidonoyl amine	C_25_H_43_NO_2_	2.8	−0.48
Glucosamine	C_6_H_13_NO_5_	2.4	0.14
Mundoserone	C_19_H_18_O_6_	2.4	−0.25
18-bromo-17E-octadecenoic acid	C_18_H_21_BrO_2_	2.1	0.06
Methotimeprazine	C_19_H_24_N_2_OS	0.3	1.15
CRACE F29			
1α, 25- dihydroxy Vitamin D3	C_27_H_44_O_3_	20.7	0.47
5-β-androstan-3a-ol-17-one sulfate	C_19_H_30_O_5_S	20.5	0.38
Proglumide	C_18_H_26_N_2_O_4_	11.9	0.80
Grayanotoxin 1	C_22_H_36_O_7_	5.4	−0.39
Promazine sulfoxide	C_17_H_20_N_2_OS	4.8	0.98
Estradiol diacetate	C_22_H_28_O_4_	4.3	0.79
6,3′-dimethoxyflavone	C_17_H_14_O_4_	3.9	0.47
Ergoline-8-methanol,10-methoxy-6-methyl-(8b)	C_17_H_22_N_2_O_2_	3.8	−0.28
N-(4-benzenesulfonamide) arachidonoyl amine	C_26_H_38_N_2_O_3_S	3.2	0.08
Desmethyl trimipramine glucuronide	C_25_H_32_N_2_O_6_	2.9	1.07
12α hydroxyl-5-deoxyhdromunduserone	C_19_H_18_O_6_	2.6	−0.25
Ranitidine	C_13_H_22_N_4_O_3_S	2.5	1.00
N-(5-hydroxy-pentyl)arachidonoyl amine	C_25_H_43_NO_2_	2.3	−0.36
Norethynodrel	C_20_ H_26_ O_2_	2.2	1.24
Praziquantel	C_19_H_24_N_2_O_2_	2.2	0.95
9,12-octadecadienal	C_18_H_32_O	2.1	−1.27
11-amino undecanoic acid	C_11_H_23_NO_2_	1.7	0.61
Amorolfine	C_21_H_35_NO	1.6	0.93
Mebhydrolin	C_19_H_20_N_2_	1.3	1.29

**Fig. 6:**
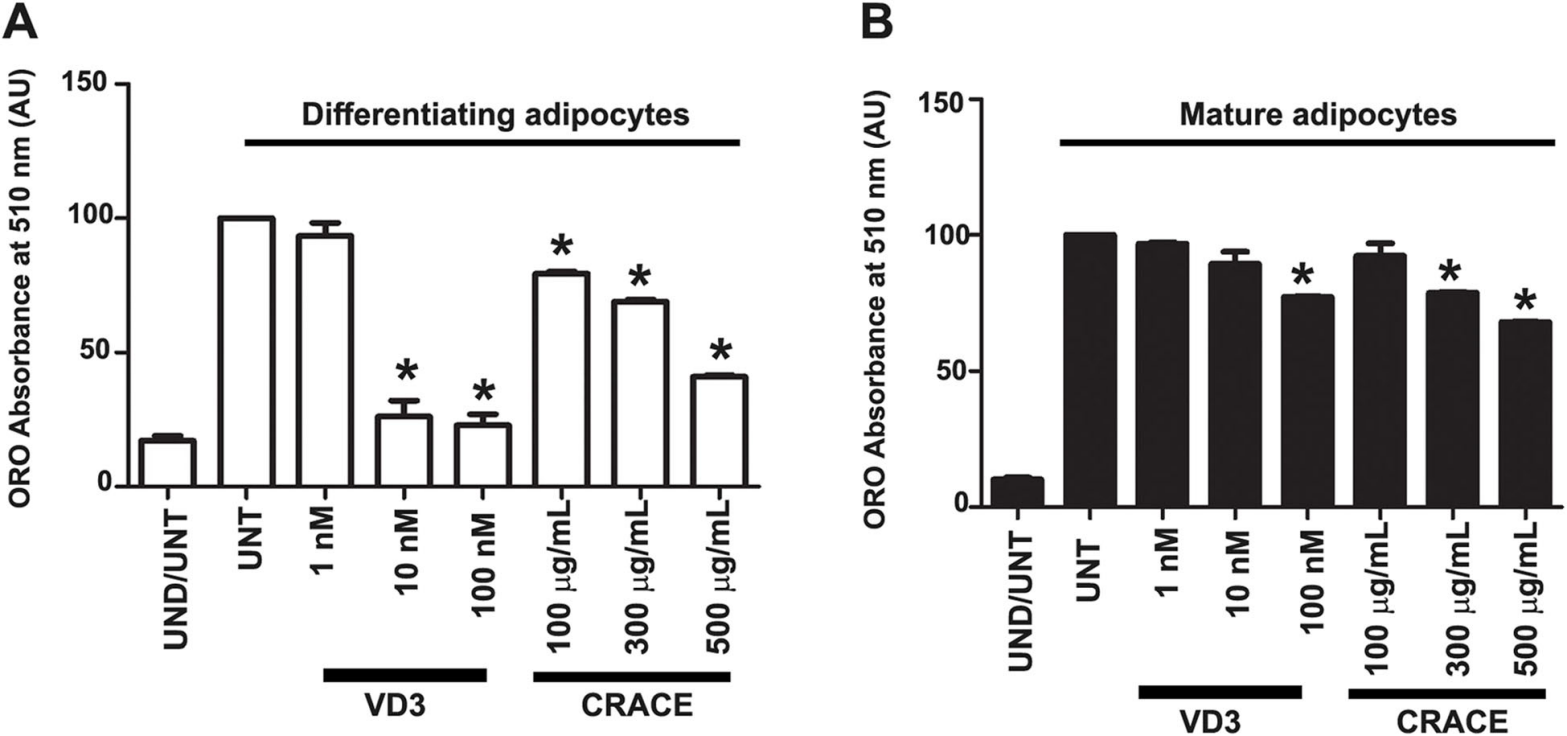
Comparison of anti-adipogenic activity of CRACE and pure 1α, 25-dihydroxy vitamin D3. **a**. Inhibition of preadipocyte differentiation by different doses of CRACE and 1α, 25-dihydroxy vitamin D3 (VD3). **b**. Treatment of differentiated mature adipocytes with different doses of CRACE and 1α, 25-dihydroxy vitamin D3 reduced lipid accumulation in these cells. In both the experiments 500 μg/mL CRACE had similar effect that of 100 nM pure 1α, 25-dihydroxy vitamin D3. Bars represent mean ± SEM, *n* = 3. One-way ANOVA followed by Bonferroni post-test was performed to evaluate the statistical significance. * indicates *p* < 0.05 for induced-untreated (UNT) vs. induced-treated

To compare the activity of CRACE with pure 1α, 25-dihydroxy vitamin D3, different doses of each of the materials were tested on 3T3-L1 differentiation and maturation. In both the assays 500 μg/mL of CRACE treatment showed a fairly equivalent effect of 100 nM (41.6 ng/mL) 1α, 25-dihydroxy vitamin D3 treatment (Fig. 6a and b).

## Discussion

Our quest for an anti-obesity therapeutic agent, lead us to investigate the medicinal herb *C. roseus*, which had well acclaimed and documented activity against cancer, diabetes and liver diseases. This study showed for the first time that the *C. roseus* aqueous crude extract (CRACE), prepared by a new method, was nontoxic to all tested cell types and had a potent anti-adipogenic effect (Fig. 1a, b and Additional file 1: Figure S1). The extract as whole, and the active fractions of the extract, displayed important dual activity; inhibition of 3T3-L1 preadipocyte differentiation, and the reduction of fat accumulation in differentiated mature adipocytes. While the extract reduced the expression of few important lipogenic genes, it concurrently, induced more than two-fold lipolysis, leading to reduced fat accumulation in mature adipocytes. Thus the extract can possibly modulate both, hypertrophy and hyperplasia, which are the predominant defects of adipocytes in obese conditions. The active fractions of the extract contain 1α, 25-dihydroxy vitamin D3 as one of the major compounds (Fig. 5) along with significant amounts of a few other molecules. It is well documented that 1α, 25-dihydroxy vitamin D3 is the biologically active form of vitamin D3 and has potent anti-adipogenic and pro-lipolytic activity on mouse cells [49–52]. However, the anti-adipogenic activity of vitamin D3 is currently inconclusive, as it exhibits rather contradictory effects on human and rat adipocytes [53–55]. A comparison of CRACE and pure 1α, 25-dihydroxy vitamin D3 activity suggests that 500 μg of the extract may contain ~ 40 ng of 1α, 25-dihydroxy vitamin D3.

Study on mechanism of CRACE activity indicated that it is a complex extract, exhibiting a range of activities. While CRACE treatment in differentiating preadipocytes has significantly inhibited expression of key adipogenic regulators PPARγ and CEBPα; the expression of these genes in mature adipocytes were not equally inhibited. Expression of PPARγ target genes showed high correlation with PPARγ and CEBPα expression level in these two conditions. While PPARγ targets like PLN1, FABP4, LPL, ATGL, and GLUT4 were highly downregulated in differentiating adipocytes (Fig. 1f), many of these genes like PLN1, FABP4, ATGL, were not downregulated in mature adipocytes (Fig. 4). The PPARγ target PLN1 is the lipid droplet coat protein, which is essential for lipid droplets formation and protection from lipases, when there are no catabolic signals [56]. Other PPARγ targets like FABP4, ATGL, and LPL play major roles in lipid metabolism and lipid uptake [23, 43]. Thus inhibition of these genes in differentiating preadipocytes essentially inhibited lipid accumulation and differentiation of these cells into adipocytes.

On the other hand, CRACE mediated reduction of lipid accumulation in mature adipocytes occurred in a different way. CRACE reduced lipid accumulation by inducing lipolysis in mature adipocytes (Fig. 4). This was achieved by increased level of cAMP, leading to PKA activation. This was confirmed from the observation that treatment of cells with PKA inhibitor H89 inhibited CRACE induced lipolysis. Earlier studies showed that 1α, 25-dihydroxy vitamin D3 treatment increases cAMP level and PKA activation in muscle cells [57]. This suggests that observed effect of CRACE on cAMP/PKA is mediated by 1α, 25-dihydroxy vitamin D3. Intriguingly CRACE reduced active Akt (p-Akt at S473) in mature adipocytes. Active Akt negatively regulates lipolysis by activating phosphodiesterase leading to reduced cAMP level [58]. Thus, decrease in p-Akt in CRACE treated cells might also have contributed in increasing the level of cAMP. Earlier studies suggested that 1α, 25-dihydroxy vitamin D3 had no effect on Akt phosphorylation in 3T3-L1 cells in normal glucose condition [59]. This in turn suggests that, there may be compounds other than the 1α, 25-dihydroxy vitamin D3, which are also part of active component of the extract. Consequently, the other molecules present in significant amounts in the active fractions, may assume this role. PKA dependent phosphorylation of major lipolytic enzyme HSL and its regulator PLN1 play a crucial positive regulatory role in the lipolytic pathway. PLN1 phosphorylation is also required for the activity of the first lipolytic enzyme ATGL which breaks down TAG. CRACE certainly stimulated a substantive PLN1 phosphorylation due to activation of PKA (Fig. 4d). CRACE did not affect the mRNA expression of the most of the lipolytic genes with exception of G0S2 which is a negative regulator of ATGL. CRACE has also reduced the expression of several lipogenic regulatory genes (SREBP1c, FAS), and lipogenic enzymes (FAS and GPD1) which contribute to de novo lipid synthesis and lipid droplet formation (Fig. 4a and b). Thus reduced lipid accumulation in mature adipocytes was a consequence of increased lipid breakdown and possibly reduced lipid production.

In differentiating preadipocytes, target of CRACE lies possibly upstream of PPARγ. CRACE clearly did not affect the expression of most of the positive (CEBPβ, CEBPδ and KLF5), or the negative (GATA2 and GATA3) regulators of PPARγ gene expression, tested at the early stage of differentiation (2 h post induction). While CRACE did affect the expression of a positive regulator Lipin1 and a negative regulator KLF7 (Fig. 2a), but it could not directly down regulate KLF7. The latter is known for its reduction of PPARγ expression and is downregulated during induction of adipogenic differentiation [34]. On the other hand, the expression of Lipin1 which strengthens the expression of CEBPα and PPARγ [60], was inhibited by CRACE immediately after induction of differentiation, paving the path for inhibition of PPARγ and CEBPα in the later stage of differentiation (as tested after 8 days of induction) (Fig. 2b). Noticeably, Lipin1 was not inhibited by CRACE in mature adipocytes (Fig. 4a). This possibly explains why there was a very small decrease observed in PPARγ and CEBPα level in CRACE treated mature adipocytes (Fig. 4a). Other adipogenic genes like SREBP1c and FAS which are involved in lipid biosynthesis and are activators of PPARγ, were also downregulated at the later stage of differentiation. Intriguingly, expression of GATA2 which was suppressed at the early stage of induction, seemed to be restored at the later stage in CRACE treated cells, possibly due to loss of other adipogenic signals (Fig. 2a and b). Earlier reports showed that anti-adipogenic action of 1α, 25-dihydroxy vitamin D3 is mediated by suppression of PPARγ, CEBPα, and SREBP1c gene expressions [61]. CRACE has also exhibited similar effects on these genes. 1α, 25-dihydroxy vitamin D3 mediated suppression of preadipocyte differentiation, happens at the early stage of differentiation, just after mitotic clonal expansion phase, but it cannot inhibit differentiation at the later stage [61]. CRACE has also modulated early gene expressions during preadipocyte differentiation.

## Conclusions

In conclusion, CRACE and its active fractions can potentially modulate adipose tissue by a two prong method; inhibiting adipogenesis as well as reducing lipid accumulation in mature adipocytes. Earlier reports on mice, indicated inhibition of adipogenesis and lipogenesis; consequently the induction of lipolysis can improve obesity and systemic glucose homeostasis [8]. CRACE has many of these properties and the additional, yet to fully explored potential, for an excellent anti-obesity therapeutic agent. Such versatile activity of CRACE can be due to presence of 1α, 25-dihydroxy vitamin D3 which may work along with other compounds of the active fractions to bring about anti-adipogenic effects [49]. This work invites future initiatives into understanding the active fractions of the extract and their effect in vivo.

## Supplementary information

**Additional file 1: Table S1.** Phytochemical analysis of CRACE. **Table S2.** Primer sequences. **Figure S1.** CRACE was non-cytotoxic to a number of tested cells at tested doses.

## Data Availability

All data generated or analysed during this study are included in this published article and its supplementary information files.

## Abbreviations

2-NBDG: (2-(N-(7-Nitrobenz-2-oxa-1,3-diazol-4-yl)Amino)-2-Deoxyglucose)
A + AD: Adenosine + Adenosine deaminase
ABHD5: α/β hydrolase domain containing protein 5
ACLY: ATP citrate lyase; AdPLA2 Adipose phospholipase A2
ATGL: Adipose triglyceride lipase
C/EBP: CAAT enhancer binding protein
DAG: Diacyl glycerol
FABP4: Fatty acid binding protein 4
FAS: Fatty acid synthase
G0S2: G0/G1 Switch gene2
GAPDH: Glyceraldehyde 3-phosphate dehydrogenase
GATA2/3: GATA binding protein 2//3
GLUT4: Glucose transporter 4
GPD: Glycerol 3-phosphate dehydrogenase
HSL: Hormone sensitive lipase
IBMX: 3-Isobutyl-1-methyl xanthine
ISO: Isoproterenol
KLF: Kruppel like factor
LPL: Lipoprotein lipase
ORO: Oil red O
PLN.1: Perilipin 1
PPARγ: Peroxisome proliferative activated receptor γ
SREBP1c: Sterol regulatory element binding protein 1c
TAG: Triacylglycerol
