# Supplementary material for: 1α, 25-dihydroxy Vitamin D3 containing fractions of Catharanthus roseus leaf aqueous extract inhibit preadipocyte differentiation and induce lipolysis in 3T3-L1 cells
Source: BMC Complement Altern Med. 2019 Nov 29;19:338. doi: 10.1186/s12906-019-2754-7 (PMC6883588; doi:10.1186/s12906-019-2754-7)
Supplement: Supplementary file 1 — Additional file 1: Table S1. Phytochemical analysis of CRACE. Table S2. Primer sequences. Figure S1. CRACE was non-cytotoxic to a number of tested cells at tested doses. [file 12906_2019_2754_MOESM1_ESM.docx]

**Title:**

1α, 25-dihydroxy Vitamin D3 containing fractions of *Catharanthus roseus* leaf aqueous extract inhibit preadipocyte differentiation and induce lipolysis in 3T3-L1 cells

**Author information:**

Anuj Kumar Borah, Archana Singh, Rafika Yasmin, Robin Doley, Venkata Satish Kumar Mattaparthi, and Sougata Saha^*^.

* Correspondence: sougata.saha@bt.nitdgp.ac.in, sougatasaha2009@gmail.com

**----------------------------------------------------------------------------------------------------------------**

**Supplementary Material**

**Table S1.** Phytochemical analysis of CRACE.

| **Test Name** | **Test For** | **CRACE** |
| --- | --- | --- |
| Foam test | Saponins | +ve |
| Phosphomolybdic Acid test | Phenolic | +ve |
| Ferric chloride test | Phenolic | +ve |
| NaOH test | Flavonoids | +ve |
| Shinoda test | Flavonoids | -ve |
| Zn-HCl test | Flavonoids | -ve |
| Braemer’s test | Tannins | +ve |
| Salkowski test | Steroids | -ve |
| Fehling test | Reducing Sugars | +ve |
| Iodine test | Non-reducing sugars | -ve |
| Bradford test | Proteins | +ve |
| Total phenolic content/ mg of extract CRACE | 54.6 µg equivalent of Gallic acid | |
| Total flavonoid content/ mg of extract CRACE | 26.5 µg equivalent of Quercetin | |
| Total tannin content /mg of extract CRACE | 392.619 µg equivalent of Tannic acid | |

**Table S2.** Primer sequences.

| **Primer** | **Primer sequence 5’-3’** | **Gene Sequence ID** | **Amplicon Size (bp)** |
| --- | --- | --- | --- |
| PPARγ1 F | TCACGTTCTGACAGGACT | NM_001127330.2 | 283 |
| PPARγ1 R | CCATTGGGTCAGCTCTTGTG |  |  |
| PPARγ2 F | GCACTGCCTATGAGCACTTC | NM_011146.3 | 211 |
| PPARγ2 R | CCATTGGGTCAGCTCTTGTG |  |  |
| C/EBP β F | CAAGCTGAGCGACGAGTACA | NM_009883.4 | 157 |
| C/EBP β R | CAGCTGCTCCACCTTCTTCT |  |  |
| C/EBP δ F | CGCAGACAGTGGTGAGCTTG | NM_007679.4 | 193 |
| C/EBP δ F | CTTGCGCACAGCGATGTTGTT |  |  |
| C/EBP α F | GTGGACAAGAACAGCAACGAG | NM-007678.3 | 223 |
| C/EBP α R | TGACCAAGGAGCTCTCAG |  |  |
| FABP4 F | CCCAACATGATCATCAGCGTAAA | NM_024406.2 | 193 |
| FABP4 R | TCGACTTTCCATCCCACTTCT |  |  |
| PLN.1 F | GGCCAAAGAGACAGCAGAATATG | NM_175640.2 | 237 |
| PLN.1 R | GTTTGCATGGTGTGTCGAGAA |  |  |
| GLUT4 F | CCAGTATGTTGCGGATGCTATG | AB008453.1 | 213 |
| GLUT4 R | TCATTCTCATCTGGCCCTAAGT |  |  |
| LPL F | GGCCCAGCAACATTATCCAG | NM008509 | 224 |
| LPL R | ACTCAAAGTTAGGCCCAGCT |  |  |
| ATGL F | ATCCCTCCTTCAACCTGGTG | AY731699 | 224 |
| ATGL R | GAGGGTAGGAGGAATGAGGC |  |  |
| HSL F | AGGACAGGACAGCAAGGTAC | NM_010719 | 169 |
| HSL R | GTTCTTGAGGTAGGGCTCGT |  |  |
| FAS F | TCTGTGCCCGTCGTCTATAC | NM-007988 | 187 |
| FAS R | GGAGGTATGCTCGCTTCTCT |  |  |
| LIPIN 1 F | ACAACAGCAGCTCCTCTACT | NM_172950.3 | 268 |
| LIPIN 1 R | CGTGCTCTTCATCACTGGAG |  |  |
| GPD F | ACTGAGATCATCAACACTCA | NM_010271.2 | 214 |
| GPD R | CCCCCTTAATAAGAGATATG |  |  |
| G0S2 F | TAGTGAAGCTATACGTGCTG | NM_008059.3 | 157 |
| G0S2 R | AGGGACTGCTGTTCACAC |  |  |
| ABHD5 F | GGGTTAAGTCTAGTGCAGCG | NM_026179.2 | 233 |
| ABHD5 R | GATCGGGCTCCAAAGATCAC |  |  |
| ACLY F | TGGTCTATTCAATTTCTACG | NM_001199296.1 | 155 |
| ACLY R | GAACTCTATATCACCCCACT |  |  |
| KLF5 F | ACTGGCCTCTACAAATCCCA | NM_009769.4 | 298 |
| KLF5 R | TCGAACTGGGCATGTCTAGA |  |  |
| KLF7 F | CAGTGACCTCATTAACGCCG | AF338369.1 | 244 |
| KLF7 R | TTTTCAGGGCATGTGTCAGC |  |  |
| GATA3 F | GAGGTGACTGCGGACCAG | NM_008091.3 | 234 |
| GATA3 R | GTGGTGGGTCGGAGGATAC |  |  |
| GATA2 F | ATGTCCAGCAAATCCAAGAAGAG | NM-008090.5 | 161 |
| GATA2 R | AGGATGTGTCCAGAGTGACTAAA |  |  |
| Adiponectin F | AAGGACAAGGCCGTTCTCT | NM_009605.5 | 219 |
| Adiponectin R | TATGGGTAGTTGCAGTCAGTTGG |  |  |
| AdPLA2 F | AAAGAACTGCTGTGCCATGT | BC024581.1 | 207 |
| AdPLA2 R | GACCTGATCACTCCGAGGAA |  |  |
| GAPDH F | CGACTTCAACAGCAACTCCCACTCTTCC | GU214026.1 | 174 |
| GAPDH R | TGGGTGGTCCAGGGTTTCTTACTCCTT |  |  |

**N.B.** For designing of primer against PPAR γ1 PPAR γ2 and GAPDH complete mRNA sequences of the respective transcripts were used. For rest of the genes being assayed CDS of corresponding gene was utilized to design the primers.

**Supplementary figures:**


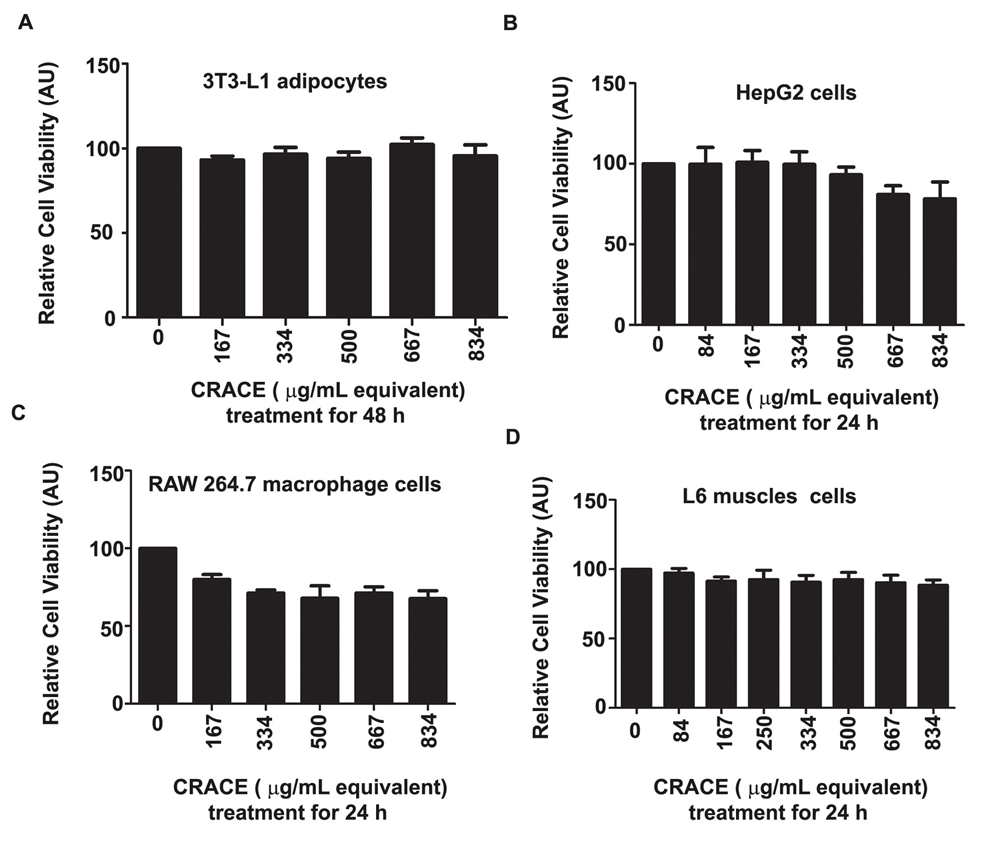


**Figure S1. CRACE was non-cytotoxic to a number of tested cells at tested doses. A.**  CRACE was non-toxic to mature 3T3-L1 adipocytes at tested dose of 167 µg/ml to 834 µg/ml equivalents during 48 h treatment. **B.** CRACE did not affect cell survivability of cultured HepG2 hepatocytes. CRACE exposure of mentioned doses for 24h did no harm the hepatocytes. **C.** 24h CRACE treatment at tested doses was non-cytotoxic to RAW 264.7 macrophages. **D.** CRACE at tested doses was non-cytotoxic to L6 rat muscle cells. All cytotoxicity results were from MTT assay. Values in the bar diagram are mean ± SEM, *n* = 3.
